# Efficacy and toxicity of thirteen plant leaf acetone extracts used in ethnoveterinary medicine in South Africa on egg hatching and larval development of *Haemonchus contortus*

**DOI:** 10.1186/1746-6148-9-38

**Published:** 2013-02-26

**Authors:** Mathew Adamu, Vinasan Naidoo, Jacobus N Eloff

**Affiliations:** 1 Phytomedicine Programme, Department of Paraclinical Sciences, Faculty of Veterinary Science, University of Pretoria, Pretoria, South Africa; 2 Department of Veterinary Parasitology and Entomology College of Veterinary Medicine, University of Agriculture Makurdi, Makurdi, Nigeria; 3 UPBRC, Faculty of Veterinary Science, University of Pretoria, Pretoria, South Africa

**Keywords:** Anthelmintic, Ethnoveterinary, Plant species, *Haemonchus contortus*, *In vitro*, Toxicity

## Abstract

**Background:**

Helminthiasis is a major limitation to the livestock industry in Africa. *Haemonchus contortus* is the singular most important helminth responsible for major economic losses in small ruminants. The high cost of anthelmintics to small farmers, resistance to available anthelmintics and residue problems in meat and milk consumed by humans further complicates matters. The use of plants and plant extracts as a possible source of new anthelmintics has received more interest in the last decade. Our aim was not to confirm the traditional use, but rather to determine activity of extracts.

Based on our past experience acetone was used as extractant. Because it is cheaper and more reproducible to evaluate the activity of plant extracts, than doing animal studies, the activity of acetone leaf extracts of thirteen plant species used traditionally in ethnoveterinary medicine in South Africa were determined using the egg hatch assay and the larval development test. Cytotoxicity of these extracts was also evaluated using the MTT cellular assay.

**Results:**

Extracts of three plant species i.e. *Heteromorpha trifoliata, Maesa lanceolata* and *Leucosidea sericea* had EC_50_ values of 0.62 mg/ml, 0.72 mg/ml and 1.08 mg/ml respectively for the egg hatch assay. *Clausena anisata*; (1.08 mg/ml) and *Clerodendrum glabrum;* (1.48 mg/ml) extracts were also active. In the larval development assay the *H. trifoliata* extract was the most effective with an EC_50_ of 0.64 mg/ml followed by *L. sericea* (1.27 mg/ml). The activities in the larval development test were generally lower in most plant species than the egg hatch assay. Based on the cytotoxicity results *C. anisata* was the least toxic with an LC_50_ of 0.17 mg/ml, while *Cyathea dregei* was the most toxic plant with an LC_50_ of 0.003 mg/ml. The C*. anisata* extract had the best selectivity index with a value of 0.10 and 0.08 for the two assays, followed by *H. trifoliata* and *L. sericea* with values of 0.07, 0.07 and 0.05, 0.04. The *C. dregei* extract had the worst selectivity index with a value of 0.00019 for both assays.

**Conclusion:**

The result of this study indicates which species should be further investigated in depth for isolation of compounds.

## Background

The parasitic gastroenteritis (PGE) complex is a disease entity caused by helminth parasites belonging to different genera mostly within the class nematoda. Of these, *Haemonchus contortus* is the single most important constraint to sheep production in South Africa [1] with estimated direct and indirect economic losses of US $45 million (I.G. Horak personal communication quoted by Waller [2]). Direct losses are due to a drop in production (carcass quality and carcass weight) or death of animals; while indirect losses are due to the costs of drugs, labour and drenching equipment required in control strategies [3,4]. The cost of controlling helminth infestation in livestock is also very high globally with chemotherapy remaining the most widely used method of treatment.

Unfortunately the excessive use of these drugs when not necessary, in addition to their use at incorrect doses, has resulted in the wide scale emergence of resistance in this parasite. Resistance of *Haemonchus contortus* was first reported in South Africa in 1975, in the benzimidazole group of anthelmintics [5], and was soon followed by successive reports of resistance to the different classes of anthelmintics [6-10]. The scope of resistance is of major concern when multi-drug resistance *Haemonchus* species no longer responded to the five major anthelmintic groups i.e. benzimidazole, ganglion blockers, macrocyclic lactones, cholinesterase inhibitors and the uncouplers of oxidative phosphorylation [11-13].

Unfortunately the situation is not getting any better and may not improve in the near foreseeable future with the result that alternative anthelmintic control options need to be developed [14]. Options tried are vaccine development, which while effective, has been bedevilled by antigenic complexity of the parasites [15]. Biological control through the use of the nematode trapping fungus, *Duddingtonia flagrans*, although once again effective, is very complicated to use as it is a pasture treatment mechanism [16,17]. Therefore the management of the parasites within the animal through the use of medication seems to be the best option. One avenue in which these new treatment agents may be discovered would be to evaluate plant extracts for their ability to treat helminth infestation [18-20].

With South Africa being rich in plant vascular flora, contributing over 10% of the world vascular flora species [21], these plants may contain chemicals that could manage resistant *Haemonchus* species [22]. More importantly in addition to an extract being active, these plants could lead to the discovery of new chemical skeletons that could be further enhanced in the laboratory. The aim of this study was to determine the *in vitro* activity of extracts of plants that are traditionally used to treat helminthic infections. This would be followed up by the isolation of bioactive compounds from the most active plant extracts.

## Methods

### Plant collection

The plants evaluated were selected based on published traditional anthelmintic use of the species in South Africa (Table 1). Leaves of the thirteen plant species were collected in November 2009 at the Pretoria National Botanical Garden where the trees were identified and labelled. Voucher specimens were made and stored in the HGW Schweickert Herbarium of the University of Pretoria. The leaves were subsequently dried at room temperature in a ventilated room, milled to a fine powder in Macsalab Mill (Model 2000 LAB Eriez®) and stored in closed containers in the dark until used.

**Table 1:** List of plant species used in the investigation, their traditional uses and references

**Plant species**	**Family**	**Medicinal uses**	**Reference**
*Brachylaena discolor*	Asteraceae (267)	Purgatives against intestinal parasites, anthelmintics for calves, sheep and goats	[23-25]
*Zanthoxylum capense*	Rutaceae (96)	Gastric and intestinal disorders,anthelmintics, cough, bronchitis,pleurisy	[23,25].
*Clerodendrum glabrum*	Lamiaceae (403)	Intestinal parasites, coughs, fever, and diabetes	[23-25].
*Heteromorpha trifoliata*	Apiaceae (491)	Intestinal worms, colic in horses and vermifuge, enemas for abdominal disorders	[23,24,26].
*Apodytes dimidiata*	Icacinaceae (139)	Enemas for intestinal parasites, purgatives, inflammation of the ear	[23,24,26].
*Strychnos mitis*	Strychnaceae (73)	Malaria, fevers	[27]
*Maesa lanceolata*	Maesaceae (615)	Anthelmintics, treatment of wounds and infertility	[24].
*Indigofera frutescens*	Papilionaceae (675)	Anthelmintics	[24].
*Leucosidea sericea*	Rosaceae (288)	Treatment of opthalmia, anthelmintics, astringents and vermifuge	[24,28].
*Melia azedarach*	Meliaceae (702)	Effective anthelmintics, emetic, cathartic and treatment of eczema	[24,25,29]
*Clausena anisata*	Rutaceae (317)	Anthelmintics, purgatives, rheumatism, fevers and myiasis	[30].
*Cyathea dregei*	Cyatheaceae (658)	Anthelmintics	[30].
*Milletia grandis*	Papilionaceae (704)	Anthelmintics and tranquilizers	[24,31]

(PRU voucher specimen numbers provided after family names).

Only the leaves of these plants species were used in the present study.

### Plant extraction

Plant material (1 g) from each species investigated was separately extracted with 10 ml of acetone, (>99% technical grade, Merck) in polyester centrifuge tubes. Acetone was selected based on its superiority as extractant based on a number of parameters [32] including that it extracts compounds with a wide range of polarities. Aqueous extracts under the conditions we used, contain only a fraction of the compounds present in acetone extracts [33,34]. We have also found that aqueous plant extracts are prone to fungal growth even when kept in a refrigerator at c.4°C possibly due to the presence of sugars or amino acids in the extracts. Furthermore drying extracts frequently led to serious complications in trying to resolubilize them.

The tube was vigorously shaken for 30 min on an orbital shaker. Tubes were centrifuged at 4000 × g for 10 min and the supernatant was filtered using Whatman No.1 filter paper before being transferred into pre-weighed glass containers. The solvent was removed by evaporation under a stream of air in a fume hood at room temperature to produce the dried extract [35]. The extract was reconstituted in 5% DMSO and tested in the assays.

### Recovery and preparation of eggs

The helminth eggs were prepared according to the method of the World Association for the Advancement of Veterinary Parasitology (WAAVP) [36] with modification. Eggs used in the study were collected from sheep with a monospecific infection of *H. contortus*. The sheep were housed indoor on concrete floor, fed hay, commercial concentrate pellets and had free access to potable water. The faecal pellets were mashed in a blender to make a relatively liquid suspension (slurry), and filtered through a 400 μm mesh sieve to remove coarse debris. Thereafter, the suspension was serially filtered through sieves of pore sizes from 250, 150, 90, 63 μm, until finally eggs were trapped on the 38 μm pore mesh. The material on the 38 μm mesh was washed into 50 ml centrifuge tubes, resuspended in a magnesium sulphate solution prepared at a specific gravity of 1.10. This was then centrifuged at 1000 × g for 10 minutes to separate the eggs from other debris. The resultant supernatant was passed through a 38 μm sieve to collect the eggs. The eggs were finally harvested by carefully washing them off the 38 μm sieve into a 1 L conical cylinder with distilled water. The concentration of eggs in an aliquot was counted under a microscope. The egg concentration was subsequently brought to a final concentration of 100 eggs per 0.2 ml.

### Egg hatch assay (EHA)

Egg hatch assay (EHA) was a modification of the WAAVP guidelines [36], using the dried plant leaf acetone extract dissolved in 5% dimethyl sulfoxide (DMSO) and albendazole as the positive control. Briefly, an aqueous egg suspension of (0.2 ml containing 100 eggs) was distributed in a 48-flat-bottomed microtitre plate and mixed with 0.2 ml of different concentrations (0.78 to 25 mg/ml) of each plant extract in 5% DMSO to give the final tested concentration of 0.39 to 12.5 mg/ml in 2.5% DMSO. Albendazole was dissolved in 5% DMSO in water and evaluated at various concentrations (0.008 to 25 μg/ml). The plates were incubated at 27°C for 48 h. After the incubation a drop of Lugol’s iodine solution was added to each well and the number of larvae and unhatched eggs were counted. The percentage inhibition of egg hatching was calculated. All experiments were undertaken in triplicate on three separate occasions (3×3). All results were compared with 5% DMSO as the negative control. In all cases eggs were subjected to 2.5% DMSO due to mixing with the same volume of aqueous suspension.

### Larval development test (LDT)

The egg suspension (100 eggs in 150 μl) was placed into 48-well plates, with 20 μl suspension of lyophilised *Escherichia coli* (ATCC 9637) [37], 10 μl amphotericin B (Sigma), 20 μl nutritive media (comprising 0.1 g yeast extract in 9 ml normal saline and 1 ml Earle’s balanced salt solution) and incubated as above for 48 h. After incubation, 200 μl of the test extracts (0.78 to 25 mg/ml) reconstituted in 5% DMSO in water and albendazole were added to the wells (n = 3) and further incubated for 5 days. Hereafter, the assay was stopped by addition of one drop of Lugol’s iodine solution and the L_1_ (first stage larvae), L_2_ (second stage larvae) and L_3_ (third stage larvae) in each well were counted using an inverted microscope. A percentage inhibition of development to L_3_ was calculated using the formula adopted from Coles et al. [36] as modified by Ademola and Eloff [38]. All results were compared to 5% DMSO as the negative control. The larvae were subjected to 2.5% DMSO because the extract was mixed with the same volume of aqueous larval suspension.

### Cytotoxicity assay using MTT

For the assay Vero monkey kidney cells obtained from a confluent monolayer cells were trypsinised and seeded (0.5 × 10^3^ cells per well) in a 96 well microtitre plate and incubated overnight at 37°C in 5% 200 μl minimal essential medium (MEM, Highveld Biological, South Africa) supplemented with 0.1% gentamicin (Virbac^R^) and 5% foetal calf serum (Adcock-Ingram). After 24 hours the media was replaced with 200 μl of the extracts (1, 0.1, 0.01, 0.001 mg/ml) and further incubated for 5 days. Viability of cells was determined using the tetrazolium-based colorimetric MTT assay (3-5-dimethyl thiazol-2-yl-2, 5-diphenyl tetrazolium bromide) described by Mosmann [39]. In short the media in each well was removed and replaced with fresh media and 30 μl of 5 mg/ml MTT in PBS and subsequently incubated for 4 h. Hereafter the medium was removed and cells washed with PBS, prior to the addition of DMSO (50 μl) to dissolve any formazan crystals present. The absorbance of the wells was measured with a Versamax microplate reader at 570 nm (pathlenght 1 cm). Different concentrations of berberine chloride (Sigma) were used as a positive control, while wells containing only cells without extracts were the negative control. The percentage cell viability relative to the pure growth was calculated. The LC_50_ values was calculated by determining the concentration of plant extracts resulting in 50% reduction of absorbance compared to untreated cells. Tests were carried out in triplicate and each experiment was repeated three times.

### Data analysis

The results generated in this study were recorded using Excel for windows 7. The EC_50_ and LC_50_ were calculated in Kinetica 5.0 (Thermo) using a sigmoid inhibitory model. The results are presented in the mean EC_50_/LC_50_ and the standard error of the mean.

## Results

### Yield

After extraction once with acetone under these conditions different yields expressed as percentage (i.e. mg extracted from 100 mg of dry material) were obtained. The yields obtained were: *L. sericea* 6.27%, *A. dimidiata* 6.07%, *Z. capense* (0.81%), *B. discolor* (3.30%), *C. glabrum* (1.60%), *H. trifoliata* (1.28%), *S. mitis* (3.75%), *M. lanceolata* (2.79%), *I. frutescens* (2.05%), *M. azedarach* (2.29%), *C. anisata* (3.40%), *C. dregei* (2.50%) and *M. grandis* (1.24%)*.*

### Egg hatch assay

**Figure 1:**
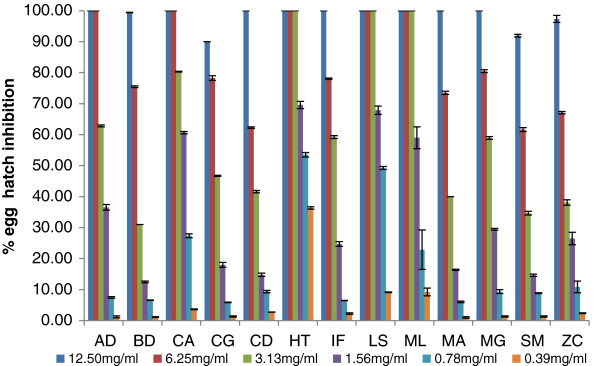
**Percentage egg hatch inhibition (mean ± SE) of different concentration of acetone leaf extracts from 13 plant species (*AD; A. dimidiata; BD;B. discolor, CA; C. anisata, CG; C. glabrum, CD; C. dregei, HT; H. trifoliata, IF; I. frutescens, LS; L. sericea, ML; M. lanceolata, MA; M. azedarach, MG; M. grandis, SM; S. mitis, ZC; Z. capense).*** Albendazole was the positive control leading to 100% inhibition even at the lowest concentration (0.008 μg/ml) used. The negative control DMSO led to < 10% inhibition.

The percentage egg hatch inhibition had a dose related response as the concentration increased (Figure 1). At the 12.5 mg/ml concentration, all the plants extracts except those from *Brachylaena discolor*, *Clerodendrum glabrum, Strychnos mitis* and *Zanthoxylum capense* inhibited egg hatching by 100%. Extracts of three plant species (*Heteromorpha trifoliata, Leucosidea sericea and Maesa lanceolata*) led to 100% inhibition at concentrations as low as 3.13 mg/ml. *H. trifoliata* had the best inhibitory activity at 0.39 mg/ml with 36.3% inhibition. The *H. trifoliata* extract had the best EC_50_ with a value of 0.62 mg/ml (Table 2), followed by *M. lanceolata* with an EC_50_ of 0.72 mg/ml. Extracts of *C. anisata, C. glabrum, A. dimidiata, B. discolor, M. grandis* and *Z. capense* had a moderate egg hatch activity with EC_50_ values ranging between 1.48-5.70 mg/ml. The plant extracts with the lowest activity were from *C. dregei* and *S. mitis* with EC_50_ of 17.64 mg/ml and 16.56 mg/ml respectively.

**Table 2:** **EC**_**50 **_**for Egg Hatch assay (EHA) and Larval Development test (LDT) with their corresponding cytotoxicity values and selectivity index (SI) for thirteen plant species**

**Names of plants**	**EHA EC**_**50**_ **± SE**	**LDT EC**_**50**_ **± SE**	**LC**_**50**_	**SI EHA**	**SI LDT**
***Apodytes dimidiata***	5.70 ± 0.23	4.13 ± 0.56	0.00396	0.000695	0.00096
***Brachylaena discolor***	3.55 ± 0.27	17.23 ± 5.47	0.00752	0.00212	0.00044
***Clausena anisata***	1.80 ± 0.09	2.07 ± 0.15	0.17186	0.09548	0.08302
***Clerodendrum glabrum***	1.48 ± 0.07	12.97 ± 2.33	0.04251	0.02872	0.00328
***Cyathea dregei***	17.64 ± 4.65	17.93 ± 6.66	0.00332	0.00019	0.00019
***Heteromorpha trifoliata***	0.62 ± 0.02	0.64 ± 0.10	0.04252	0.06858	0.06644
***Indigofera frutescens***	7.11 ± 1.10	7.58 ± 1.05	0.1044	0.01468	0.01377
***Leucosidea sericea***	1.08 ± 0.11	1.27 ± 0.07	0.0515	0.04769	0.04055
***Maesa lanceolata***	0.72 ± 0.05	1.68 ± 0.10	0.01577	0.0219	0.00939
***Melia azedarach***	6.24 ± 0.20	10.96 ± 1.79	0.14466	0.02318	0.0137
***Milletia grandis***	5.57 ± 0.33	6.11 ± 1.04	0.05336	0.00958	0.00873
***Strychnos mitis***	16.56 ± 4.88	16.94 ± 4.71	0.01721	0.00104	0.00102
***Zanthoxylum capense***	13.26 ± 0.24	13.64 ± 3.44	0.02095	0.00643	0.00153

**Note**: all values for EHA, LDT and LC_50_ in mg/ml; Albendazole was positive control and recorded 100% inhibition at all concentrations (0.008 to 25 μg/ml) used, while 2.5% DMSO recorded < 10% inhibition.

### Larval development test

**Figure 2:**
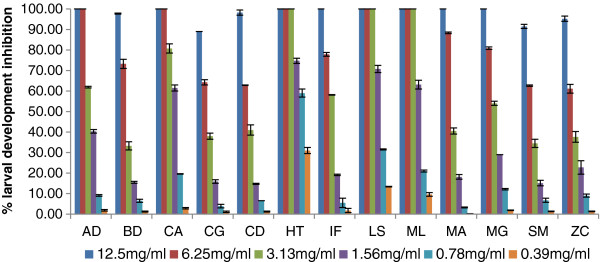
**Percentage larval development inhibition (mean ± SE) of different concentration of acetone leaf extracts from 13 plant species (*AD; A. dimidiata; BD; B. discolor, CA; C. anisata, CG; C. glabrum, CD; C. dregei, HT; H. trifoliata, IF; I. frutescens, LS; L. sericea, ML; M. lanceolata, MA; M. azedarach, MG; M. grandis, SM; S. mitis, ZC; Z. capense).*** Albendazole was the positive control leading to 100% inhibition even at the lowest concentration (0.008 μg/ml) used. The negative control 2.5% DMSO led to < 10% inhibition.

Similar to the egg hatch assay, the larval development assay had a linear dose related inhibitory response (Figure 2). At concentrations of 12.5 mg/ml extracts of 8 of the 13 plant species led to complete inhibition, while extracts from five plants (*Brachylaena discolor, Clerodendrum glabrum, Cyathea dregei, Strychnos mitis* and *Zanthoxylum capense*) had an activity less than 100%. For *H. trifoliata, L. sericea and M. lanceolata* extracts, 100% inhibition was still evident at 6.25 and 3.13 mg/ml. At the lowest concentration tested (0.39 mg/ml) *H. trifoliata* again had the highest activity with 31% inhibition. The EC_50_ of all thirteen plants acetone extracts are presented in Table 2. The EC_50_ of *H. trifoliata* was the best at 0.64 mg/ml, followed by *L. sericea* and *M. lanceolata* with 1.27 and 1.68 mg/ml respectively. Both *C. dregei* and *B. discolor* were rather ineffective with the EC_50_ values of 17.93 and 17.23 mg/ml respectively.

### Cytotoxicity

The results for the cytotoxicity using the MTT assay are shown in Table 2. *Clausena anisata* had the highest LC_50_ (lowest toxicity) of 0.17 mg/ml, followed by *Melia azedarach* at 0.14 mg/ml. *C. dregei* was highly toxic with an LC_50_ of 0.003 mg/ml. Some plant extracts were more toxic to the cells than to the helminth indicating that it would probably not be useful in treating animals. This can be expressed by calculating the selectivity index (SI) of the plant species based on both assays. The selectivity index is calculated by dividing the cytotoxicity LC_50_ to the EC_50_ of the assay. The higher the value of the selectivity index the safer the extract is. This value helps to eliminate activity that may possibly be due to a general metabolic toxin. The *C. anisata* extract was the best for both assays; this was followed by *H. trifoliata* and *L. sericea* for both assays and plant species respectively. The plant species with the worst selectivity index was *C. dregei* for both assays.

## Discussion

The main objective of this study was to establish the inhibitory activity of leaf acetone extracts of the 13 plant species traditionally used for their ability to influence the egg hatching and larval development of *Haemonchus contortus*. Acetone was selected as a suitable extractant due to its ability to extract compounds of a wide polarity range, its miscible with organic and aqueous solvents and is non-toxic to bacteria and fungi organisms [32]. Acetone is a better extractant for plant secondary compounds than water the common solvent used by rural communities. Because a different extractant was used, these results do not necessarily confirm or dispute the traditional use of plant material by rural pastoralists, as compounds of intermediate polarity may be released by microbial or photo-oxidation processes after extraction. In addition when leaves are fed or particulate material is ingested, it is usually the intermediate to lipid soluble compounds that become bioavailable due to the influence of bile salts or the gastrointestinal tract mucosal barrier preventing the absorption of water soluble compounds [40].

For the evaluation of the results we propose that extracts with EC_50_ above 6 mg/ml should be considered to have weak anthelmintic activity as it may be difficult to achieve such high concentration *in vivo*. When this criterion is applied to the results, the EHA indicate *H. trifoliata, L. sericea and M. lanceolata*, with EC_50s_ from 0.62-1.08 mg/ml as potential candidates for further isolation work. These extracts also recorded 100% egg hatch inhibition within the highest concentrations of 12.5, 6.25 and 3.13 mg/ml. While the actives are unknown at this stage, the activity recorded in this study may be attributed to secondary metabolites [41]. The activity shown by *M. lanceolata* in the present study agrees with earlier work that evaluated the anthelmintic property of various parts of this plant on *H. contortus* from Asia [42]. This activity may be due to presence of saponins in the leaf of the plant [43]. Saponins are known to destabilize cell membranes hence increase cell permeability by combining with membranes associated sterols [44].

*Leucosidea sericea* extracts had a reasonably good EC_50_ value (1.08 mg/ml) in the current study, thus agreeing with a recent study by Aremu et al. [45] who reported a minimum lethal concentration of 0.52 mg/ml for the petroleum ether leaf extract using *Caenorhabditis elegans*. The difference in EC_50_ values may be attributed to the differences in susceptibility between the non-pathogenic free living *C. elegans* used in their study compared to the pathogenic *H. contortus* we used. The activity of the extract of *L. sericea* may be due to aspidinol [46], condensed tannins or alkaloids [45]. Condensed tannins have anthelmintic activity with varied possible mechanism of actions [47] most especially its astringent property. This is the first report of good anthelmintic activity of *Heteromorpha trifoliata* and it may be associated with compounds such as fulcarindiol and sarison [48] with antifungal activity from the leaves of this plant. This plant has been used as a vermifuge in horses, by the Xhosa people of South Africa.

The larval development inhibition test yielded similar results to the EHA, *H. trifoliata, L. sericea* and *M. lanceolata*, once again had excellent activity*.* Most plants extracts had weak activity with EC_50_ of 7 mg/ml and above. The activity of *M. lanceolata* extract confirms activity by Tadesse et al. [42] on the same species from a different origin (Ethiopia). The plants extracts generally had better inhibition activity on the eggs than on the larva of *H. contortus* based on the EC_50_ values recorded. This is contrary to report by Ademola and Eloff [22] where they had better activity for the larval inhibition compared to the egg hatch assay. The result of this study may be significant as the inhibition of egg hatch is possibly an important method of reducing pasture contamination by the animals during grazing helping in the overall helminth control programme.

In comparison to work undertaken using extracts of other plant species, the activity shown by the three plants with the best activity were in the same order of activities found by Bizimenyera et al., [49] using *Peltophorum africanum* leaf, bark and roots as well as extracts of *Coriandum sativum* on eggs and larval development of *H. contortus*[50]. Bizimenyera et al., [49] recorded an EC_50_ of 0.62 mg/ml and 0.72 mg/ml for the leaf acetone extract for the EHA and LDT assays respectively. This is in the same order with values of 0.62 and 0.64 mg/ml recorded in the EHA and LDT for *H. trifoliata* in the current study. The EC_50_ of *H. trifoliata* was lower than the acetone leaf extracts of *Combretum molle* 0.87 mg/ml and 0.60 mg/ml for the EHA and LDT respectively [22]. The weak activity recorded by *M. azedarach* (EC_50_ = 10.96 mg/ml) agrees with Maciel et al., [51] in Brazil were they reported an LC_50_ of 9.18 mg/ml with the leaf ethanol extract of the plant. It is encouraging that despite the difference in geographical location and organic extractants used, similar bioactivity was obtained for related species.

While the activity from this study show the potential value of plant extracts in the management of haemonchosis. The results need to be interpreted with caution as *in vitro* activity may not automatically translate into *in vivo* efficacy. Factors that still need to be considered are animal factors such as absorption and presystemic elimination [52]. More importantly the potential toxicity of the molecule needs to be considered. For this study we used renal cells in culture as an indicator of toxicity. The cells were specifically selected as the kidney is one of the main sites of excretion in animals due to the preferential blood supply of the kidney in addition to their high metabolic capacity. The results of the cytotoxicity study were disappointing as in all cases the extracts were toxic to the cells.

However the presence of cellular toxicity is not conclusive as several factors interplay with toxicity results. In this case, it is possible that the use of an organic extractant may have led to extraction of toxic compounds. For this chemical fractionation and isolation may help separate out the potential toxic compounds. It is also possible that *in vivo* interaction with microsomal and non-microsomal pathways may render the molecule less toxic due to metabolism or simply gut-barrier exclusion may play a role. As a result it is suggested that the three most efficacious plants, which had lower toxicity profiles, be evaluated for further study by fractionation and possibly using a dedicated animal model.

Our experience was that aqueous extracts contained few compounds, had very low biological activity [33,34] and had low or negligible anthelmintic activity [49,53]*.* Many other authors have also used organic extracts in evaluating anthelmintic activity [20,38,49]. The way some traditional healers use water extracts by storing it for some time may lead to the solubilisation of intermediate polarity compounds by microbial action or photo-oxidation of compounds in the extract. The aqueous extraction carried out in the laboratory does not approximate the traditionally used methods. In work that was completed after this study had started, members in our group decided to use laboratory aqueous extraction conditions on a plant with excellent antifungal activities [54]. To our surprise the aqueous extract of *Markhamia obtusifolia* had double the activity of the acetone extract and it was slightly less toxic to Vero kidney cells than to *Haemonchus contortus*[54]. These results indicate that we should determine the anthelminthic activity of aqueous extracts of plant species that we have already investigated.

In future studies water extracts should therefore also be evaluated for activity and safety. Our policy to date was to extract at room temperature to limit possible changes in the metabolites at high temperatures. To get closer to the conditions used traditionally boiling water extracts and storing under different conditions should also be investigated.

There is also the possibility of using the plant material in the feed directly to control helminths. In an experiment to feed *Cereus jamacara* an invasive plant species grazed by game and used by a commercial farmer to control helminths had only partial success in decreasing helminth load [55].

## Conclusion

Although direct comparisons may be difficult because traditional healers mainly use water as an extractant it does appear as if several of these traditionally used plants are active against *Haemonchus* infections. Although the activity of all extracts was several orders of magnitude lower than that of the positive control, high concentrations may be attainable in the animal gut if there are no toxicity problems. It is useful to determine the *in vivo* toxicity of promising extracts to determine if cellular toxicity is a good indicator of *in vivo* toxicity. The most promising plants extracts for further study are *M. lanceolata, L. sericea* and *H. trifoliata*. Because substantial work has been done on *M. lanceolata* we have fractionated the extracts of *L.sericea* and have isolated bioactive compounds and tested their activity *in vitro*. *In vivo* animal study will also be evaluated using the acetone extracts of *L. sericea* in a sheep model.

## Competing interests

There are no conflicts of interest.

## Authors’ contributions

MA participated in the design of the study, carried out field work, prepared the extracts, participated in all assay, analysed the data and wrote first draft and subsequent drafts of the manuscript. VN participated in the design and coordination of the study, supervised the study, analysed the data and revised the draft manuscript. JNE participated in the design and coordination of the study, supervised the study and revised the manuscript. All authors read and approved the final manuscript.
